# The saturation effect of a body shape index on lumbar bone mineral density in US adults: Findings from a nationwide survey

**DOI:** 10.1371/journal.pone.0324160

**Published:** 2025-06-02

**Authors:** Ziyi Zhao, Hongxiang Ji, Wenyu Liu, Zhengdan Wang, Shengquan Ren, Chunlei Liu, Caifeng Wu, Jian Wang, Xiaoheng Ding

**Affiliations:** 1 Department of Hand and Foot, Microsurgery, The Affiliated Hospital of Qingdao University, Qingdao, Shandong, China; 2 The First Clinical Medical College, Shandong University of Traditional Chinese Medicine, Jinan, Shandong, China; City College of New York, UNITED STATES OF AMERICA

## Abstract

**Background:**

Many studies have demonstrated that obesity is closely linked with bone metabolism. A body shape index (ABSI) is a newly developed obesity indicator, which provides superior reflection of central obesity compared to body mass index (BMI) and waist circumference. Nevertheless, investigation of the association between ABSI and bone mineral density (BMD) remains limited. Thus, this study aimed to evaluate the correlation between ABSI and lumbar BMD among US adults.

**Methods:**

We analyzed data of adults aged 20 years and older from 2011–2018 National Health and Nutrition Examination Survey. Weighted multiple regression analysis was conducted to assess the linear relationship between ABSI and lumbar BMD. Weighted smooth curve fitting and two-segment linear model were applied to explore the nonlinear association. Subgroup analysis stratified by age, gender, race, and BMI was performed.

**Results:**

A total of 10991 subjects was enrolled in the study. In the fully adjusted model, the ABSI was negatively related to lumbar BMD (β = −0.007, 95% CI: −0.009, −0.005). The adverse correlation remained significant across all subgroups among stratified analysis. The saturation effect between ABSI and lumbar BMD was identified, with the turning point at the ABSI value of 0.08. Similar nonlinear trends were also observed in participants aged <40 years, males, Non-Hispanic White, BMI < 25 kg/m2, and BMI ≥ 30 kg/m2.

**Conclusion:**

This study revealed a prominently negative correlation and saturation effect between ABSI and lumbar BMD in US adults. Our findings may provide valuable inspiration for further prevention and intervention of osteoporosis.

## Introduction

As a systemic bone disease, osteoporosis (OP) is characterized by bone microstructure degradation and reduced bone mineral density (BMD) [1,2]. It is estimated that over 70 million people in the US will be afflicted with bone loss or OP by 2030 [3]. The medical expenditure on OP is expected to increase from $57 billion in 2018 to more than $95 billion each year in 2040 in the US [4]. Johnell et al. report over 8.9 million fractures owing to OP per year worldwide [5]. Thus, OP has become a noteworthy public health problem, and the development of an objective, effective, and convenient strategy to early detect and further prevent OP has drawn ever-growing attention.

Obesity is characterized by excessive or anomalous body fat, which has a detrimental effect on health and is highly related to several chronic disorders [6]. Its prevalence has been prominently increasing with approximate morbidity of 30% worldwide [7,8]. Numerous researches have demonstrated the close association between obesity and bone health. Investigating this relationship via anthropometric index is essential. While body mass index (BMI) and waist circumference (WC) act as traditional obesity indictors, muscle and fat mass cannot be discriminated by these metrics [9–11]. Krakauer et al. proposed a body shape index (ABSI) as a novel obesity measure in 2012 by taking WC, weight, and height into account. The ABSI could reliably indicate visceral deposition of adipose tissue, and suggest abdominal fat accumulation, which provides superior reflection of central obesity compared to BMI and WC [12,13]. The ABSI is highly associated with various diseases, including metabolic syndrome [14], diabetes [15,16], cancer [17,18], and cardiovascular diseases [19–21]. Moreover, the ABSI demonstrates better predictive ability than both BMI and WC in abdominal aortic calcification [22], hypertension [23], and mortality [24,25].

To date, while few studies have explored the relationship between ABSI and bone health, current evidence is still limited, which requires further investigation. The aim of this study was to assess the correlation between ABSI and lumbar BMD among US adults aged 20 years and older by analyzing the 2011–2018 National Health and Nutrition Examination Survey (NHANES) database.

## Methods

### Data source and study population

NHANES, conducted by the National Center for Health Statistics (NCHS), is the largest population-based national cross-sectional survey worldwide. The nutritional and health condition of noninstitutionalized US population is examined by this survey via applying a complex, stratified, multistage sampling approach. Now, NHANES collects and releases data in 2-year cycles.

**Fig 1:**
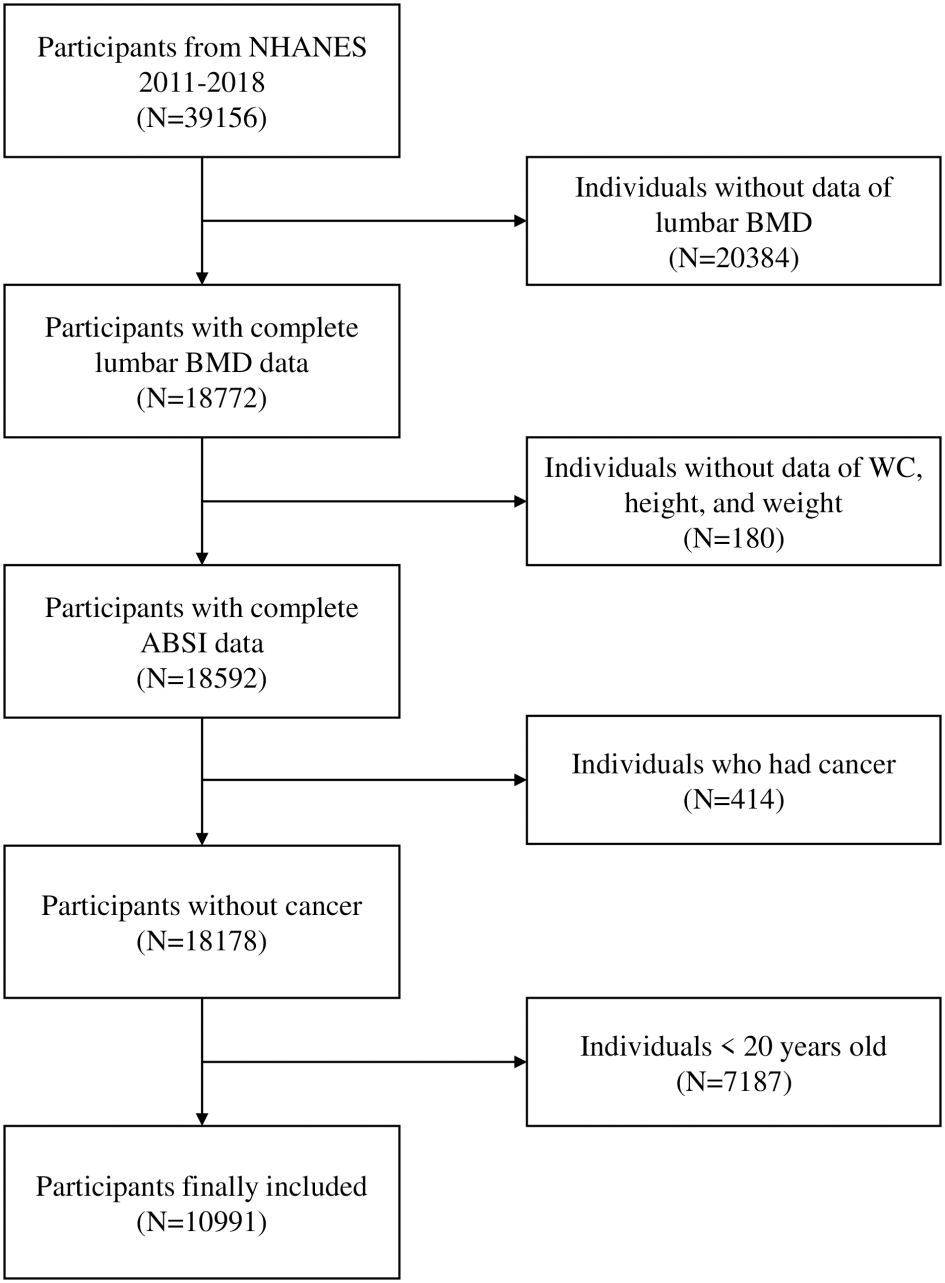
Flowchart of participants selection. Abbreviation: NHANES, National Health and Nutrition Examination Survey; BMD, bone mineral density; WC, waist circumference; ABSI, a body shape index.

Our data consisted of four cycles of NHANES (2011–2018). Of a total 39,156 subjects, 20384 individuals with missing lumbar BMD data, 180 individuals without available data of WC, height, and weight, 414 individuals suffering from cancer, and 7187 individuals aged < 20 years were excluded. Ultimately, the data of 10991 participants were retained in the final analysis (Fig 1). Ethics Review Board of the NCHS ratified the NHANES protocol, and informed consent was provided by each individual.

### ABSI evaluation

ABSI acted as the independent variable in this study, and the formula below was used to determine ABSI [26]:


$$ABSI=WC\times {height}^{5/6}\times {weight}^{-2/3}=\frac{WC}{{height}^{1/2}\times {BMI}^{2/3}}$$

Well-trained professionals performed all the body measurements in the mobile examination facility. To determine the WC, a measuring tape was placed at the junction of two lines, which were drawn at the right midaxillary line and horizontally above the uppermost lateral border of the right iliac bone, respectively. The height was recorded in standard standing position and the body weight was evaluated by calibrated platform scale for barefoot subjects with light clothes.

### Outcome variable

As the dependent variable, lumbar BMD was examined by dual-energy x-ray absorptiometry (DXA) applying Hologic Discovery model A densitometers (Hologic, Inc., Bedford, Massachusetts) and Apex 3.2 software. Qualified radiologists conducted all examinations.

### Covariates

Covariates included: demographics data [age, sex, race, education, ratio of family income to poverty (PIR)], examination data (BMI, WC, height, weight), questionnaire data (smoking status, alcohol use, moderate and vigorous activities, diabetes, hypertension), laboratory data [serum glucose, alanine aminotransferase (ALT), aspartate aminotransferase (AST), alkaline phosphatase (ALP), total protein, albumin, serum creatinine, serum uric acid, blood urea nitrogen (BUN), phosphorus, total calcium, triglyceride, total cholesterol, high-density lipoprotein cholesterol (HDL-C), low-density lipoprotein cholesterol (LDL-C)], dietary data (protein intake, calcium intake).

Weight (kg) was divided by square of height (m) to calculate BMI. Subjects who have smoked at least 100 cigarettes were defined as smokers. Alcohol use status included 5 categories: never (<12 drinks in lifetime), former (≥12 drinks in lifetime or any past year and without alcohol intake last year), mild (females having ≥1 drinks/day, males having ≥2 drinks/day), moderate (females having ≥2 drinks/day, males having ≥3 drinks/day, or binge drinking ≥2 days/month), heavy (females having ≥3 drinks/day, males having ≥4 drinks/day, or binge drinking ≥5 days/month) [27]. Diabetes and hypertension were evaluated by subject’s self-reported doctor diagnosis. Additional details of all covariates are accessible at www.cdc.gov/nchs/nhanes/.

### Statistical analysis

Data were analyzed by operating R (version 4.3) and Empowerstats (version 4.1). Appropriate sample weights were applied in accordance with NCHS analytical guideline to ensure the representation of national population [28,29]. Continuous and categorical variables were expressed as Mean (SE) and n (%) respectively. Weighted chi-square test and one-way analysis of variance were used to evaluate the characteristics of population by ABSI quartile. The relationship between ABSI and lumbar BMD was examined by performing weighted multiple regression analysis. ABSI was further categorized into quartiles for trend test. Three models were constructed: model 1 adjusted for no covariate; model 2 adjusted for age, gender, and race; and model 3 adjusted for all covariates except height, weight, BMI and WC. Additionally, stratified analysis and interaction test were carried out in fully adjusted model. The nonlinear association between ABSI and lumbar BMD was investigated by using weighted smooth curve fitting and two-segment linear model. P-value <0.05 was considered statistically significant.

### Ethics approval and consent to participate

Ethics Review Board of the NCHS ratified the NHANES protocol, and informed consent was provided by each individual.

## Results

### Characteristics of study participants

The characteristics of participants are presented according to the ABSI quartiles (Table 1). In general, this study enrolled 10991 subjects, with a mean (SE) age of 39.01 (0.23) years and 52.86% of male participants. The mean (SE) values of ABSI and lumbar BMD among all individuals were 0.08 (0.00) and 1.04 (0.00) g/cm2, respectively. Compared with subjects in the bottom ABSI quartile, those in the top quartile were more likely to be older, male, Non-Hispanic White, and Mexican American, demonstrated higher values of PIR, BMI, WC, weight, height, smoking rate, serum glucose, ALT, AST, ALP, uric acid, BUN, triglyceride, total cholesterol, and LDL-C, and showed greater prevalences of diabetes and hypertension. In contrast, they exhibited lower education level, alcohol use level, physical activity, total protein, albumin, phosphorus, HDL-C, protein intake, and lumbar BMD. There existed no significant between-group difference among creatinine, total calcium, and calcium intake.

**Table 1:** Weighted characteristics of the study population based on a body shape index quartile.

Variables	Total N = 10991	A body shape index				P-value
		Q1 (0.058–0.077) N = 2511	Q2 (0.078–0.080) N = 2724	Q3 (0.081–0.083) N = 2884	Q4 (0.084–0.099) N = 2872	
Age (years)	39.011 (0.229)	33.219 (0.401)	37.014 (0.343)	40.234 (0.265)	44.363 (0.267)	< 0.0001
Sex, n (%)						< 0.0001
Female	5298 (47.139)	1320 (55.244)	1325 (47.154)	1262 (41.744)	1391 (45.985)	
Male	5693 (52.861)	1191 (44.756)	1399 (52.846)	1622 (58.256)	1481 (54.015)	
Race/ethnicity, n (%)						< 0.0001
Non-Hispanic White	3679 (60.122)	684 (52.719)	881 (59.129)	988 (61.394)	1126 (65.777)	
Non-Hispanic Black	2487 (12.190)	914 (21.633)	609 (12.025)	504 (9.047)	460 (7.865)	
Mexican American	1604 (10.411)	231 (7.463)	408 (10.892)	503 (12.218)	462 (10.524)	
Other Race	3221 (17.277)	682 (18.185)	826 (17.954)	889 (17.341)	824 (15.835)	
Education, n (%)						< 0.0001
Less than high school	2040 (13.464)	334 (9.638)	472 (12.166)	585 (14.721)	649 (16.529)	
High school	2415 (21.982)	525 (20.463)	595 (21.399)	611 (21.520)	684 (24.229)	
More than high school	6534 (64.543)	1652 (69.898)	1655 (66.392)	1688 (63.759)	1539 (59.242)	
Unknow	2 (0.011)	0 (0.000)	2 (0.044)	0 (0.000)	0 (0.000)	
PIR	2.937 (0.050)	2.844 (0.076)	2.976 (0.061)	3.039 (0.064)	2.871 (0.070)	0.01
Waist circumference (cm)	98.142 (0.356)	88.469 (0.427)	94.676 (0.408)	100.408 (0.476)	106.983 (0.486)	< 0.0001
Height (cm)	169.635 (0.166)	168.100 (0.241)	169.541 (0.265)	170.374 (0.304)	170.225 (0.297)	< 0.0001
Weight (kg)	83.559 (0.409)	79.150 (0.592)	81.830 (0.558)	85.250 (0.623)	87.070 (0.653)	< 0.0001
BMI (kg/m2)	28.962 (0.142)	28.076 (0.220)	28.394 (0.184)	29.263 (0.215)	29.916 (0.213)	< 0.0001
Smoking status, n (%)						< 0.0001
Yes	4263 (40.561)	808 (32.441)	984 (36.113)	1196 (43.977)	1275 (47.923)	
No	6723 (59.414)	1702 (67.536)	1740 (63.887)	1684 (55.948)	1597 (52.077)	
Unknow	5 (0.025)	1 (0.023)	0 (0.000)	4 (0.075)	0 (0.000)	
Alcohol use, n (%)						< 0.0001
Never	1337 (9.232)	307 (10.307)	317 (8.272)	312 (7.859)	401 (10.654)	
Former	890 (7.356)	145 (5.434)	171 (5.575)	261 (7.410)	313 (10.548)	
Mild	3312 (31.509)	804 (30.221)	837 (33.358)	869 (32.030)	802 (30.271)	
Moderate	1863 (18.820)	458 (20.746)	488 (17.782)	486 (20.517)	431 (16.544)	
Heavy	2617 (25.512)	596 (26.009)	675 (26.984)	698 (25.113)	648 (24.112)	
Unknow	972 (7.571)	201 (7.282)	236 (8.030)	258 (7.071)	277 (7.871)	
Moderate activities, n (%)						< 0.001
Yes	7180 (69.903)	1742 (72.868)	1799 (69.738)	1888 (71.239)	1751 (66.319)	
No	3811 (30.097)	769 (27.132)	925 (30.262)	996 (28.761)	1121 (33.681)	
Vigorous activities, n (%)						< 0.0001
Yes	5263 (51.228)	1484 (62.192)	1422 (56.033)	1306 (48.704)	1051 (40.332)	
No	5728 (48.772)	1027 (37.808)	1302 (43.967)	1578 (51.296)	1821 (59.668)	
Serum glucose (mmol/L)	5.394 (0.026)	5.028 (0.027)	5.190 (0.034)	5.468 (0.046)	5.805 (0.059)	< 0.0001
ALT (u/L)	26.297 (0.245)	22.374 (0.447)	25.906 (0.487)	27.488 (0.458)	28.613 (0.530)	< 0.0001
AST (u/L)	25.243 (0.195)	23.993 (0.282)	24.794 (0.357)	25.482 (0.395)	26.431 (0.566)	< 0.0001
ALP (u/L)	67.107 (0.424)	63.059 (0.587)	64.495 (0.577)	67.385 (0.617)	72.551 (0.791)	< 0.0001
Total protein (g/L)	71.445 (0.110)	71.511 (0.159)	71.561 (0.151)	71.558 (0.144)	71.166 (0.126)	0.036
Albumin (g/L)	43.195 (0.074)	43.327 (0.115)	43.517 (0.099)	43.350 (0.106)	42.626 (0.110)	< 0.0001
Creatinine (umol/L)	75.901 (0.320)	76.701 (0.485)	76.000 (0.533)	76.130 (0.480)	74.934 (0.616)	0.139
Uric acid (umol/L)	319.449 (1.293)	302.190 (2.134)	319.157 (1.943)	325.815 (2.249)	327.142 (2.230)	< 0.0001
BUN (mmol/L)	4.580 (0.028)	4.464 (0.042)	4.634 (0.050)	4.585 (0.043)	4.616 (0.043)	0.011
Phosphorus (mmol/L)	1.198 (0.003)	1.211 (0.005)	1.199 (0.005)	1.191 (0.005)	1.195 (0.005)	0.026
Total calcium (mmol/L)	2.343 (0.002)	2.345 (0.003)	2.344 (0.002)	2.343 (0.003)	2.339 (0.003)	0.328
Triglyceride (mmol/L)	1.360 (0.027)	1.026 (0.033)	1.320 (0.054)	1.499 (0.048)	1.519 (0.037)	< 0.0001
Total cholesterol (mmol/L)	4.941 (0.018)	4.632 (0.028)	4.914 (0.028)	5.042 (0.025)	5.110 (0.029)	< 0.0001
Direct HDL-C (mmol/L)	1.360 (0.008)	1.461 (0.013)	1.375 (0.014)	1.336 (0.012)	1.289 (0.010)	< 0.0001
LDL-C (mmol/L)	2.951 (0.016)	2.676 (0.031)	2.945 (0.035)	3.056 (0.031)	3.069 (0.033)	< 0.0001
Diabetes, n (%)						< 0.0001
Yes	781 (5.535)	66 (2.414)	117 (2.888)	229 (5.967)	369 (10.140)	
No	10005 (92.756)	2411 (96.487)	2569 (95.834)	2604 (92.637)	2421 (86.935)	
Borderline	198 (1.664)	34 (1.099)	35 (1.224)	50 (1.387)	79 (2.815)	
Unknow	7 (0.045)	0 (0.000)	3 (0.054)	1 (0.008)	3 (0.110)	
Hypertension, n (%)						< 0.0001
Yes	2557 (22.036)	400 (14.344)	527 (18.525)	729 (23.897)	901 (29.724)	
No	8424 (77.904)	2109 (85.562)	2193 (81.390)	2154 (76.083)	1968 (70.228)	
Unknow	10 (0.060)	2 (0.095)	4 (0.085)	1 (0.020)	3 (0.049)	
Protein intake (g)	87.620 (0.621)	89.680 (1.522)	89.423 (0.815)	86.944 (1.050)	84.954 (1.167)	0.006
Calcium intake (mg)	1013.397 (9.414)	1029.612 (19.840)	1033.219 (16.344)	1005.715 (13.662)	989.506 (16.190)	0.178
Lumbar BMD (g/cm2)	1.040 (0.002)	1.094 (0.005)	1.052 (0.003)	1.024 (0.003)	1.000 (0.004)	< 0.0001

Mean (SE) for continuous variables: the p-value was calculated by the weighted one-way analysis of variance. n (%) for categorical variables: the p-value was calculated by the weighted chi-square test.

Abbreviation: Q, quartile; PIR, ratio of family income to poverty; BMI, body mass index; ALT, alanine aminotransferase; AST, aspartate aminotransferase; ALP, alkaline phosphatase; BUN, blood urea nitrogen; HDL-C, high-density lipoprotein cholesterol; LDL-C, low-density lipoprotein cholesterol; BMD, bone mineral density.

### Linear association between ABSI and lumbar BMD

Table 2 shows the correlation between ABSI and lumbar BMD in three linear regression models. When no confounding factor was adjusted, ABSI was negatively linked with lumbar BMD in model 1 (β = −0.008, 95% CI: −0.010, −0.007). After partially and fully adjusting for covariates, this negative association still persisted in model 2 (β = −0.007, 95% CI: −0.009, −0.006) and model 3 (β = −0.007, 95% CI: −0.009, −0.005). Compared with the lowest ABSI quartile, the lumbar BMD in the highest quartile dropped by 0.074 g/cm2 (β = −0.074, 95% CI: −0.097, −0.052 and p for trend <0.0001) after transforming ABSI from a continuous variable to a classified variable (quartile).

**Table 2:** Association between a body shape index and lumbar bone mineral density among adults.

Exposure	Model 1		Model 2		Model 3	
	β (95% CI)	P-value	β (95% CI)	P-value	β (95% CI)	P-value
ABSI (per 0.001 unit increase)	−0.008 (−0.010, −0.007)	<0.0001	−0.007 (−0.009, −0.006)	<0.0001	−0.007 (−0.009, −0.005)	<0.0001
ABSI (quartile)						
Q1 (0.058–0.077)	Reference		Reference		Reference	
Q2 (0.078–0.080)	−0.043 (−0.054, −0.032)	<0.0001	−0.034 (−0.045, −0.023)	<0.0001	−0.037 (−0.054, −0.020)	<0.001
Q3 (0.081–0.083)	−0.070 (−0.083, −0.058)	<0.0001	−0.058 (−0.071, −0.045)	<0.0001	−0.057 (−0.077, −0.037)	<0.0001
Q4 (0.084–0.099)	−0.094 (−0.107, −0.081)	<0.0001	−0.081 (−0.095, −0.068)	<0.0001	−0.074 (−0.097, −0.052)	<0.0001
P for trend	< 0.0001		< 0.0001		< 0.0001	

Model 1: no covariate was adjusted.

Model 2: age, gender, and race were adjusted.

Model 3: age, gender, race, PIR, education, smoking status, alcohol use, moderate activities, vigorous activities, serum glucose, ALT, AST, ALP, total protein, albumin, creatinine, uric acid, BUN, phosphorus, total calcium, total cholesterol, triglyceride, HDL-C, LDL-C, hypertension, diabetes, protein intake, and calcium intake were adjusted.

Abbreviation: ABSI, a body shape index; Q, quartile; PIR, ratio of family income to poverty; ALT, alanine aminotransferase; AST, aspartate aminotransferase; ALP, alkaline phosphatase; BUN, blood urea nitrogen; HDL-C, high-density lipoprotein cholesterol; LDL-C, low-density lipoprotein cholesterol.

**Fig 2:**
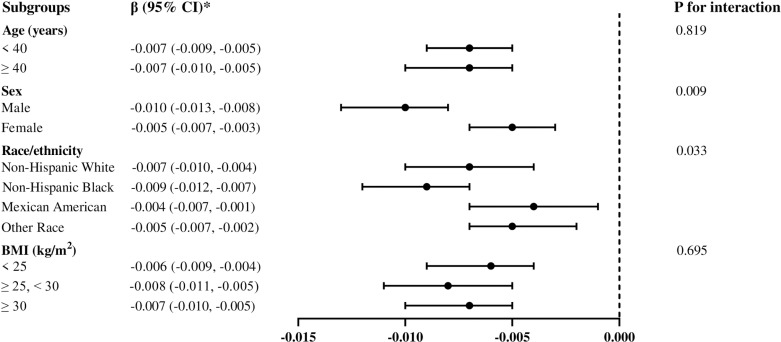
Subgroup analysis of the association between a body shape index and lumbar bone mineral density. *β (95% CI) was calculated based on per 0.001 unit increase in a body shape index. Age, gender, race, PIR, education, smoking status, alcohol use, moderate activities, vigorous activities, serum glucose, ALT, AST, ALP, total protein, albumin, creatinine, uric acid, BUN, phosphorus, total calcium, total cholesterol, triglyceride, HDL-C, LDL-C, hypertension, diabetes, protein intake, and calcium intake were adjusted, but the model was not adjusted for the stratification variables themselves. Abbreviation: BMI, body mass index; PIR, ratio of family income to poverty; ALT, alanine aminotransferase; AST, aspartate aminotransferase; ALP, alkaline phosphatase; BUN, blood urea nitrogen; HDL-C, high-density lipoprotein cholesterol; LDL-C, low-density lipoprotein cholesterol.

Stratified analysis was performed to assess the stability of the correlation between ABSI and lumbar BMD across several subgroups, including age, gender, race, and BMI (Fig 2). Higher ABSI was significantly related to lower BMD across all subgroups (all p < 0.01). The negative association was not modified by age and BMI (p for interaction >0.05). When stratified by genders, the correlation between ABSI and lumbar BMD was more prominent in male (β = −0.010, 95% CI: −0.013, −0.008) than female (β = −0.005, 95% CI: −0.007, −0.003). Among subgroup of race, the negative relationship was more significant in Non-Hispanic Black (β = −0.009, 95% CI: −0.012, −0.007) compared to Mexican American (β = −0.004, 95% CI: −0.007, −0.001).

### Nonlinear association between ABSI and lumbar BMD

**Fig 3:**
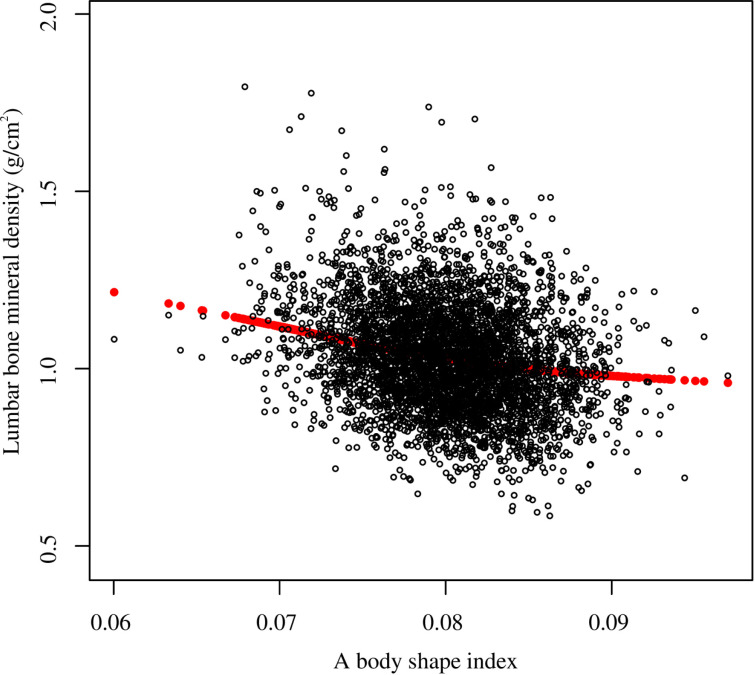
The nonlinear association between a body shape index and lumbar bone mineral density. Each black hollow point exhibits one participant. Age, gender, race, PIR, education, smoking status, alcohol use, moderate activities, vigorous activities, serum glucose, ALT, AST, ALP, total protein, albumin, creatinine, uric acid, BUN, phosphorus, total calcium, total cholesterol, triglyceride, HDL-C, LDL-C, hypertension, diabetes, protein intake, and calcium intake were adjusted. Abbreviation: PIR, ratio of family income to poverty; ALT, alanine aminotransferase; AST, aspartate aminotransferase; ALP, alkaline phosphatase; BUN, blood urea nitrogen; HDL-C, high-density lipoprotein cholesterol; LDL-C, low-density lipoprotein cholesterol.

**Fig 4:**
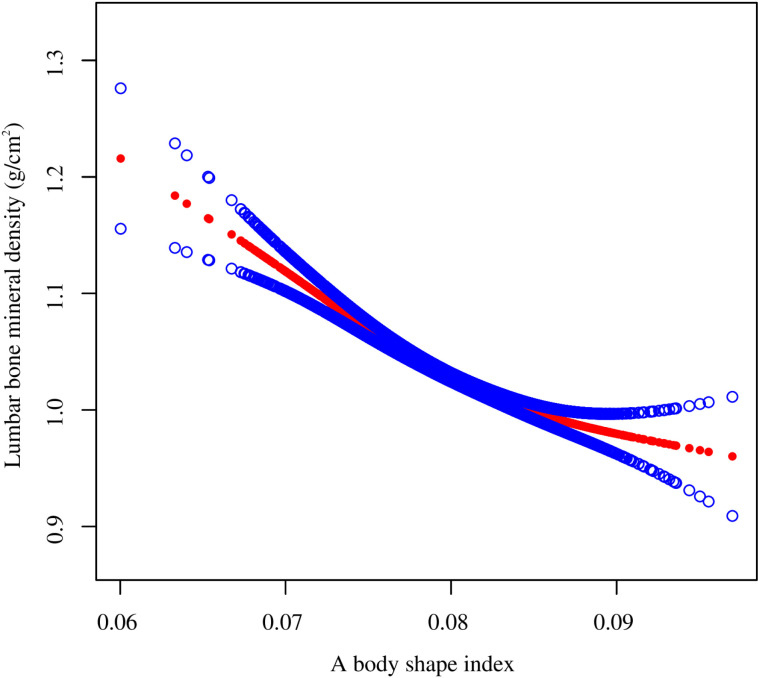
The nonlinear association between a body shape index and lumbar bone mineral density. Solid red line illustrates the fitted smooth curve among variables. Two blue bands illustrate the 95% CI of the fit. Age, gender, race, PIR, education, smoking status, alcohol use, moderate activities, vigorous activities, serum glucose, ALT, AST, ALP, total protein, albumin, creatinine, uric acid, BUN, phosphorus, total calcium, total cholesterol, triglyceride, HDL-C, LDL-C, hypertension, diabetes, protein intake, and calcium intake were adjusted. Abbreviation: PIR, ratio of family income to poverty; ALT, alanine aminotransferase; AST, aspartate aminotransferase; ALP, alkaline phosphatase; BUN, blood urea nitrogen; HDL-C, high-density lipoprotein cholesterol; LDL-C, low-density lipoprotein cholesterol.

**Fig 5:**
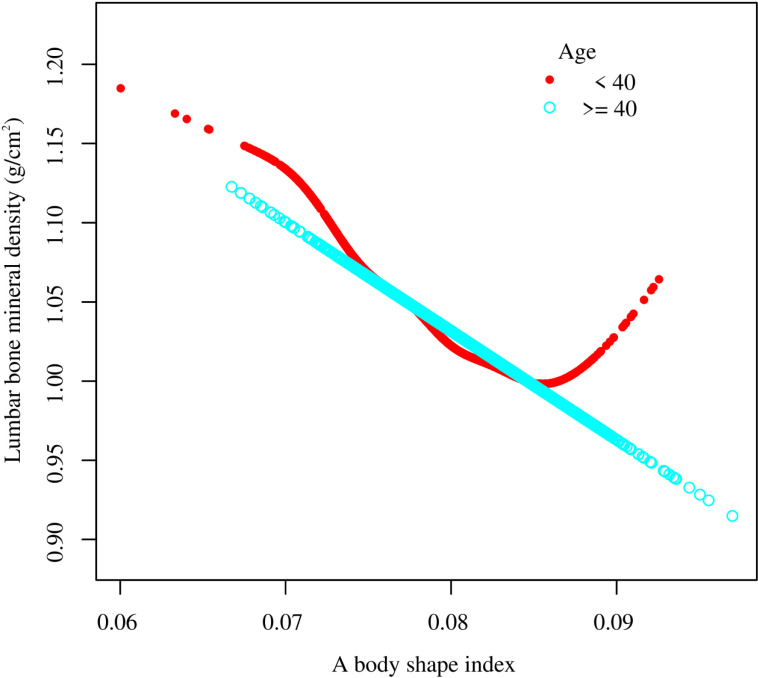
Subgroup analysis for the association between a body shape index and lumbar bone mineral density stratified by age. Age, gender, race, PIR, education, smoking status, alcohol use, moderate activities, vigorous activities, serum glucose, ALT, AST, ALP, total protein, albumin, creatinine, uric acid, BUN, phosphorus, total calcium, total cholesterol, triglyceride, HDL-C, LDL-C, hypertension, diabetes, protein intake, and calcium intake were adjusted, but the model was not adjusted for the stratification variables themselves. Abbreviation: PIR, ratio of family income to poverty; ALT, alanine aminotransferase; AST, aspartate aminotransferase; ALP, alkaline phosphatase; BUN, blood urea nitrogen; HDL-C, high-density lipoprotein cholesterol; LDL-C, low-density lipoprotein cholesterol.

**Fig 6:**
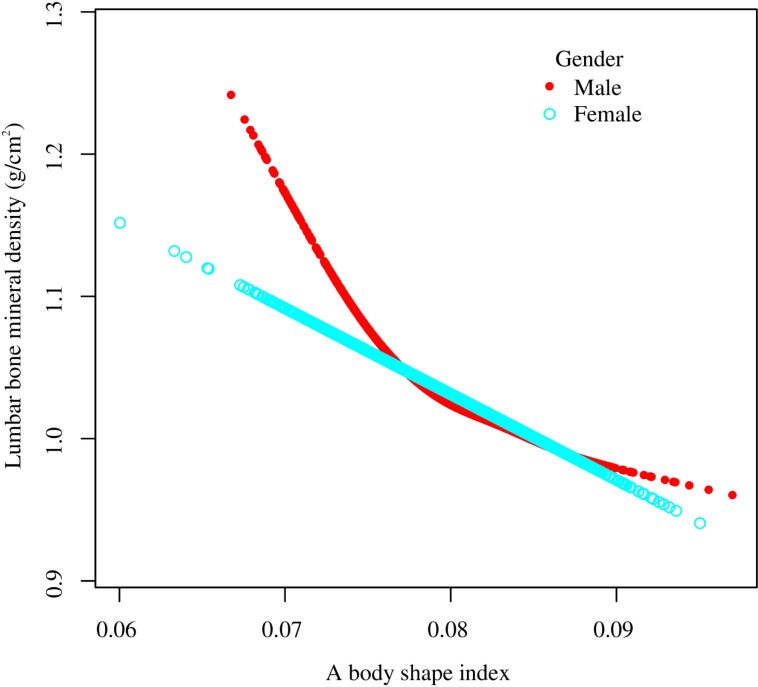
Subgroup analysis for the association between a body shape index and lumbar bone mineral density stratified by gender. Age, gender, race, PIR, education, smoking status, alcohol use, moderate activities, vigorous activities, serum glucose, ALT, AST, ALP, total protein, albumin, creatinine, uric acid, BUN, phosphorus, total calcium, total cholesterol, triglyceride, HDL-C, LDL-C, hypertension, diabetes, protein intake, and calcium intake were adjusted, but the model was not adjusted for the stratification variables themselves. Abbreviation: PIR, ratio of family income to poverty; ALT, alanine aminotransferase; AST, aspartate aminotransferase; ALP, alkaline phosphatase; BUN, blood urea nitrogen; HDL-C, high-density lipoprotein cholesterol; LDL-C, low-density lipoprotein cholesterol.

**Fig 7:**
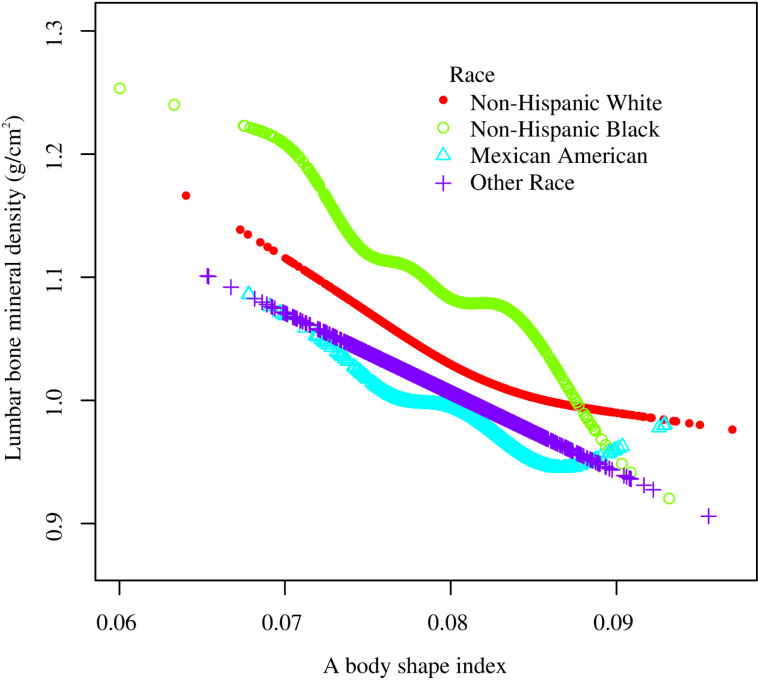
Subgroup analysis for the association between a body shape index and lumbar bone mineral density stratified by race/ethnicity. Age, gender, race, PIR, education, smoking status, alcohol use, moderate activities, vigorous activities, serum glucose, ALT, AST, ALP, total protein, albumin, creatinine, uric acid, BUN, phosphorus, total calcium, total cholesterol, triglyceride, HDL-C, LDL-C, hypertension, diabetes, protein intake, and calcium intake were adjusted, but the model was not adjusted for the stratification variables themselves. Abbreviation: PIR, ratio of family income to poverty; ALT, alanine aminotransferase; AST, aspartate aminotransferase; ALP, alkaline phosphatase; BUN, blood urea nitrogen; HDL-C, high-density lipoprotein cholesterol; LDL-C, low-density lipoprotein cholesterol.

**Fig 8:**
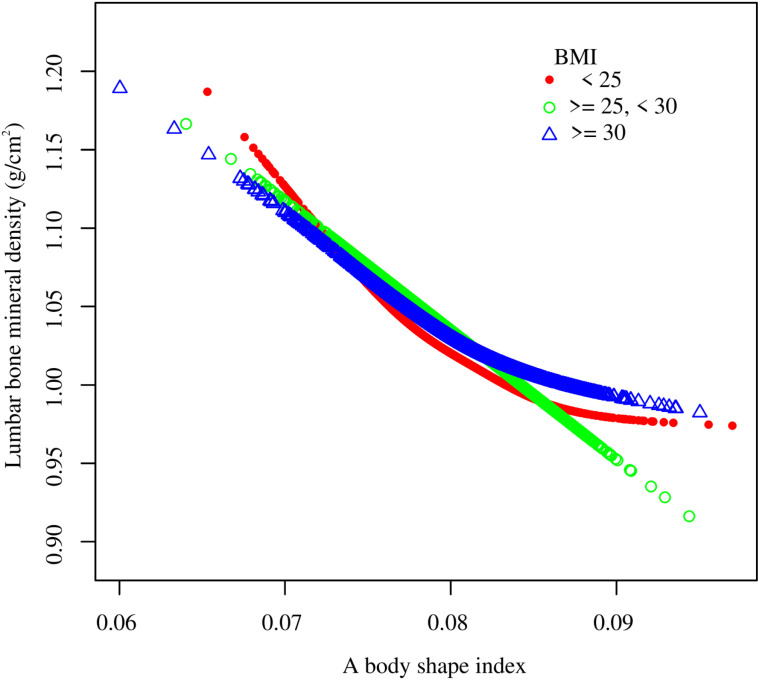
Subgroup analysis for the association between a body shape index and lumbar bone mineral density stratified by BMI. Age, gender, race, PIR, education, smoking status, alcohol use, moderate activities, vigorous activities, serum glucose, ALT, AST, ALP, total protein, albumin, creatinine, uric acid, BUN, phosphorus, total calcium, total cholesterol, triglyceride, HDL-C, LDL-C, hypertension, diabetes, protein intake, and calcium intake were adjusted, but the model was not adjusted for the stratification variables themselves. Abbreviation: BMI, body mass index; PIR, ratio of family income to poverty; ALT, alanine aminotransferase; AST, aspartate aminotransferase; ALP, alkaline phosphatase; BUN, blood urea nitrogen; HDL-C, high-density lipoprotein cholesterol; LDL-C, low-density lipoprotein cholesterol.

Smooth curve fitting revealed the saturation effect between ABSI and lumbar BMD with the saturation value of 0.08 (Fig 3, Fig 4; Table 3). For subjects with ABSI <0.08, every 0.001 unit increment in ABSI was related to a 0.01 g/cm2 decrease in lumbar BMD (95% CI: −0.012, −0.008); meanwhile, for ABSI >0.08, every 0.001 unit growth in ABSI was linked with a 0.004 g/cm2 decrease in lumbar BMD (95% CI: −0.006, −0.002). Furthermore, similar nonlinear relationship also existed in participants aged <40 years (turning point: 0.082), males (turning point: 0.079), Non-Hispanic White (turning point: 0.085), BMI < 25 kg/m2 (turning point: 0.087), and BMI ≥ 30 kg/m2 (turning point: 0.081) (Fig 5–8; Table 3).

**Table 3:** Saturation effect analysis of ABSI on lumbar bone mineral density using the two-piecewise linear regression model.

Outcome	Turning point (K)	ABSI < K	ABSI > K	Log likelihood ratio
		Adjusted β (95% CI)^*^ P value	Adjusted β (95% CI)^*^ P value	
Total	0.080	−0.010 (−0.012, −0.008) <0.0001	−0.004 (−0.006, −0.002) <0.0001	<0.001
Subgroup analysis				
Age < 40 years old	0.082	−0.009 (−0.011, −0.008) <0.0001	0.002 (−0.003, 0.006) 0.4447	<0.001
Male	0.079	−0.018 (−0.022, −0.015) <0.0001	−0.005 (−0.008, −0.003) <0.0001	<0.001
Non-Hispanic White	0.085	−0.009 (−0.011, −0.007) <0.0001	0.003 (−0.004, 0.009) 0.4590	0.004
BMI < 25 kg/m2	0.087	−0.008 (−0.010, −0.006) <0.0001	0.011 (−0.000, 0.022) 0.0608	0.002
BMI ≥ 30 kg/m2	0.081	−0.012 (−0.014, −0.009) <0.0001	−0.001 (−0.005, 0.002) 0.4789	<0.001

* β (95% CI) was calculated based on per 0.001 unit increase in ABSI.

Age, gender, race, PIR, education, smoking status, alcohol use, moderate activities, vigorous activities, serum glucose, ALT, AST, ALP, total protein, albumin, creatinine, uric acid, BUN, phosphorus, total calcium, total cholesterol, triglyceride, HDL-C, LDL-C, hypertension, diabetes, protein intake, and calcium intake were adjusted, but the model was not adjusted for the stratification variables themselves.

Abbreviation: ABSI, a body shape index; BMI, body mass index; PIR, ratio of family income to poverty; ALT, alanine aminotransferase; AST, aspartate aminotransferase; ALP, alkaline phosphatase; BUN, blood urea nitrogen; HDL-C, high-density lipoprotein cholesterol; LDL-C, low-density lipoprotein cholesterol.

## Discussion

To our knowledge, this study possesses the largest sample size for evaluating the correlation between ABSI and BMD in adults. In this cross-sectional study of 10991 US adults, we identified a negative relationship between ABSI and lumbar BMD. Similar association was also observed in stratified analysis. Meanwhile, there existed saturation effect between ABSI and lumbar BMD with the turning point of 0.08.

Kim et al. reported that ABSI was negatively correlated with bone health in 6717 middle-aged and older Korean individuals [30]. In addition, a positive relationship between ABSI and the prevalence of osteoporosis was identified in adjusted model among 2534 elderly Chinese participants [31]. Moreover, a cross-sectional study from China indicated that ABSI was positively associated with osteoporosis in 3,457 subjects [32]. Our findings are consistent with these previous researches, and further determine the saturation effect between ABSI and BMD.

Globally, BMI and WC are acknowledged anthropometric parameters for obesity assessment [33–36]. Previous studies concluded that BMI and WC were positively related to BMD. A population-based study of 6,143 US adolescents showed a positive correlation between BMI and total BMD with a saturation effect value of 21.5 kg/m2 [37]. Song et al. conducted a Mendelian randomization study by applying single nucleotide polymorphisms highly correlated with BMI among 336,107 subjects and reported that BMI causally increased heel calcaneus BMD and lumbar BMD [38]. A meta-analysis revealed that the obesity, defined by BMI, was positively associated with BMD in lumbar spine and femoral neck [39]. Likewise, Alay et al. indicated a significantly positive relationship between BMI, WC, femoral neck BMD, and L1-L4 lumbar spine BMD by analyzing data from 452 postmenopausal women in Turkey [40]. A cross-sectional study based on 2,903 older adults aged ≥50 years from 2017–2020 NHANES demonstrated that the BMI and WC were positively linked with femoral neck BMD with a BMI saturation value of 24.3 kg/m2 [41].

Nevertheless, when the obesity is assessed by applying BMI and WC, an obesity paradox has been reported recently by numerous researchers. The obesity paradox, as a counterintuitive phenomenon, suggests that obesity may provide protective effects and lead to superior prognoses in particular diseases [42,43]. It is believed by several researchers that the obesity paradox may be actually inexistent and it results from the restriction of BMI in distinguishing fat mass from muscle mass [44–46]. This paradox has casted doubt on the reliability of WC and BMI acting as obesity evaluation indices in scholars [47–50]. To further investigate the correlation between adiposity and bone health, Jiao et al. determined body composition by DXA and identified a negative association between total percent fat and BMD in 11,615 Americans aged 18 years and older [51]. Our findings demonstrate that increase in ABSI is significantly associated with decrease in lumbar BMD and this negative relationship remains constant across subgroups, which are consistent with this research and distinct from previous studies defining obesity by BMI and WC. Moreover, a cross-sectional study showed that ABSI possessed better discriminatory ability in osteoporosis than BMI and WC among 130 kidney transplant recipients from Turkey [52]. Therefore, ABSI might be a preferable parameter to assess obesity status.

In addition, obesity and sarcopenia frequently coexist in clinical populations [53,54]. Cheng et al. indicated that lumbar BMD is negatively associated with sarcopenia in US adults based on NHANES database [55]. Similar outcomes were reported in both older women and patients with type 2 diabetes [56,57].

The mechanisms of the detrimental correlation between obesity and bone health are unclear. There are several possible mechanisms: First, while adiponectin promotes the differentiation of bone mesenchymal stem cells (BMSCs) in bone marrow to osteoblasts via CXCL1 and CXCL8 up-regulation, subjects with obesity are prone to obtain lower levels of adiponectin [58,59]. Second, mainly secreted by white adipose tissue, leptin shows a dual effect upon bone tissue. On the one hand, it is reported that leptin enhances the differentiation of stromal cells to osteoblasts and inhibits the formation of osteoclast in vitro researches [60,61]. Moreover, lower femoral BMD and bone volume are observed among leptin gene knockout rats [62]. On the other hand, there is a negative association between leptin and serotonin from neurons in hypothalamus, resulting in damage to bone formation [63]. Generally, the leptin exhibits a dominantly detrimental effect on bone health [6,64]. Astudillo et al. discover that obese participants tend to gain elevated leptin levels [60]. Third, increased secondary hyperparathyroidism prevalence is correlated with obesity with characteristic of high parathyroid hormone level, thereby leading to low BMD [65]. Forth, obesity enhances the quantity of adipocytes among the bone marrow and regulates their metabolism. Both adipocytes and osteoblasts are differentiated from BMSCs among the bone marrow [66,67]. The obesity facilitates BMSCs to differentiate into adipocytes, which contributes to the replacement of osteoblasts by adipocytes within the bone marrow [68]. The excessive buildup of adipocytes in the bone marrow leads to imbalanced osteocyte activity and low bone turnover, which may culminate in the early occurrence of OP [69]. Finally, obesity is related to elevated susceptibility to inflammation [70]. In the microenvironment of bone marrow, the increase in adipocytes limits osteoblast differentiation, hinders osteoprotegerin release, promotes the formation and activation of osteoclasts, and accelerates the secretion of inflammatory and immunoregulatory substances which stimulate the production of osteoclasts [71,72].

Undeniably, this study has several limitations. First, causality between ABSI and lumbar BMD in adults was unable to be determined in this study due to the application of cross-sectional methodology. Second, it was possible that additional cofounding parameters failed to be fully taken into account, for example, the treatment of osteoporosis. Finally, the current findings were not generalizable for subjects with cancer as this population was excluded. Despite these restrictions, it is noteworthy that the large sample size representative of US population is used in this study, making it possible to perform subgroup analysis. Furthermore, conducting longitudinal studies in the future is essential for confirming our findings.

## Conclusion

The ABSI acted as a negative predictor for bone health in US adults. The saturation effect between ABSI and lumbar BMD was observed. Our findings indicate that maintaining appropriate ABSI level may be essential for effective management of bone health.
